# Two-scale concurrent simulations for crack propagation using FEM–DEM bridging coupling

**DOI:** 10.1007/s40571-024-00788-x

**Published:** 2024-07-27

**Authors:** Manon Voisin-Leprince, Joaquin Garcia-Suarez, Guillaume Anciaux, Jean-François Molinari

**Affiliations:** Institute of Civil Engineering, Institute of Materials Science and Engineering, École Polytechnique Fédérale de Lausanne (EPFL), 1015 Lausanne, Switzerland

**Keywords:** Finite element method, Discrete element method, Granular, Multiscale, Bridging coupling, Crack propagation

## Abstract

The Discrete element method (DEM) is a robust numerical tool for simulating crack propagation and wear in granular materials. However, the computational cost associated with DEM hinders its applicability to large domains. To address this limitation, we employ DEM to model regions experiencing crack propagation and wear, and utilize the finite element method (FEM) to model regions experiencing small deformation, thus reducing the computational burden. The two domains are linked using a FEM–DEM coupling, which considers an overlapping region where the deformation of the two domains is reconciled. We employ a “strong coupling” formulation, in which each DEM particle in the overlapping region is constrained to an equivalent position obtained by nodal interpolation in the finite element. While the coupling method has been proved capable of handling propagation of small-amplitude waves between domains, we examine in this paper its accuracy to efficiently model for material failure events. We investigate two cases of material failure in the DEM region: the first one involves mode I crack propagation, and the second one focuses on rough surfaces’ shearing leading to debris creation. For each, we consider several DEM domain sizes, representing different distances between the coupling region and the DEM undergoing inelasticity and fracture. The accuracy of the coupling approach is evaluated by comparing it with a pure DEM simulation, and the results demonstrate its effectiveness in accurately capturing the behavior of the pure DEM, regardless of the placement of the coupling region.

**Supplementary Information:**

The online version contains supplementary material available at 10.1007/s40571-024-00788-x.

## Introduction

The Discrete element method (DEM), originally developed for modeling granular materials, has been successfully applied to simulate crack propagation in dense materials [10, 12, 18, 21, 41]. Further DEM studies have focused on investigating the properties and characteristics of a third-body layer (TBL), commonly known as “gouge,” which forms when two bodies slide relative to each other in contact [1, 17, 22, 23, 36]. These studies considered no cohesion in the contact law between particles, except for [1, 23] which incorporated an initial cohesion but did not account for particle reattachment. On the other hand, other studies focused on the formation of the third-body layer through the wear formation process [26, 27, 40]. Pham-Ba and Molinari [27] modeled wear formation using DEM, with a formulation enabling attachment and reattachment of particles. This contact law enabled the authors to replicate key wear mechanisms observed in MD simulations while significantly reducing computational costs by using particles that are ten times larger than the bond lengths in MD. Mollon [26] modeled the wear formation considering cohesion between particles, but also considering the deformability of the grains. While all these methods allow for in-depth analysis of the gouge and its formation, they still remain costly and are therefore limited to small domains.

To reduce the computational cost, coupling methods can be employed to connect finite element method (FEM) with DEM. These methods are inspired by others, which were developed to couple MD with FEM. The coupling methods can be classified into two main categories: overlapping methods (FEM–MD: [35, 38], FEM–DEM: [13, 34, 37], Material Point Method-DEM: [14, 39]) and edge-to-edge methods (FEM–MD: [11, 29, 31], FEM–DEM: [9, 20, 25]). Overlapping methods offer several advantages compared to edge-to-edge methods, including reduced wave reflection [38] and lower computational cost as the continuum discretization does not require refinement down to the particle scale, resulting in savings in degrees of freedom. There are notable differences in the coupling between DEM and FEM compared to MD and FEM, especially with overlapping methods that simulate the same material in both domains. A primary challenge is ensuring material property consistency across FEM and DEM, due to the amorphous nature of the discrete domain with material properties assigned at the particle scale (Hertz contact). To address this, Wellmann and Wriggers [37] computed the bulk mechanical behavior of a fixed-size representative volume element (RVE) of DEM, and consequently fit the continuum. Subsequently, Voisin-Leprince et al. explored various RVE sizes to identify the minimum RVE size at which elastic properties converge. This minimum RVE size set the element size to use in the FEM to match material properties. Voisin-leprince et al. also explored the impact of considering non-cohesive particles, where prestress must be applied in FEM to balance the DEM. The introduction of prestress lead to ghost forces, which necessitates the implementation of effective mitigation strategies.

In their study [34], the FEM and DEM were connected using an overlapping method also known as the *Bridging method* [38], which involves a “reconciliation zone” where the discrete and continuum domains coexist Within this zone, their deformations are harmonized thanks to the *Arlequin method* [16], which considers an energy weighting of both domains, as well as kinetic constraints using Lagrange multipliers. Various forms of kinematic constraints, including weak and strong formulations, were considered. The study employed Hertz contact between the particles and focused on the transmission of small-amplitude waves. Both formulations were demonstrated to be effective, with the strong formulation exhibiting superior stability. Additionally, they showed the effect of incorporating a “force subtraction” method at the FEM–DEM interface to mitigate the influence of ghost forces [30].

In this manuscript, the previously described coupling method [34] is employed to computationally investigate two material failure events in a quasi-2D simulation box: the first case involves mode I crack propagation, while the second case involves wear leading to debris formation. We employ the discrete element method to capture both crack propagation and wear, and utilize the finite-element method to save computational resources in modeling the regions that remain linear and elastic. Both DEM and FEM represent the third-body layer and therefore are modeled with matching material properties. To model the material portion that undergoes failure, we use the coarse-grained discrete element technique developed by Pham-Ba and Molinari [28]. The FEM–DEM coupling is realized using the FEM–DEM bridging coupling developed by Voisin-Leprince et al. [34]. Section 2 presents the method used in this paper, including the coarse-grained discrete element model, the FEM–DEM bridging coupling, and the geometry of the simulations. Section 3 shows the result obtained for the two investigated cases. Section 4 provides closing remarks.

## Method

### Discrete domain

To model crack propagation and wear formation in the discrete domain, we employ the coarse-grained approach developed by Pham-Ba and Molinari [28], which enables detachment and reattachment of particles thanks to appropriate adhesive forces. The parameters of the interaction forces between particles are determined such that the assembly of several particles exhibits certain mechanical properties (see Ref. [28] and supplementary material). Therefore, employing this contact law enables controlling the macroscopic material properties exhibited by the DEM, which facilitates the matching of material properties between the FEM and DEM. Similarly to Pham-Ba and Molinari [28], we considered the macroscopic material properties of $\text {SiO}_2$ (see Table 1) for the DEM and FEM. The critical particle size required by the contact law to achieve an accurate match of macroscopic material properties is $d_{\text {c}}= \text {1.7 nm}$ (see Eq. 16 in supplementary material). The minimum particle size determined by computational constraints is $d_{\text {min}}= \text {0.37 nm}$ (see Eq. 17 in supplementary material). If a particle is smaller than this value, it will interact with more particles than its closest neighbors, increasing the computational cost. Based on these values, we chose a log normal distribution of particle sizes with a mean grain diameter of $d_{0} = \text {1.3 nm}$, a maximum grain diameter of $d_{\text {max}}=1.2d_{0}$, a minimum grain diameter of $d_{\text {min}}=0.8d_{0}$, and a variance of $0.2(d_{\text {max}}-d_{\text {min}}) \text { nm}$.

**Table 1:** Amorphous silica properties (SiO$_{2}$)

SiO$_{2}$	
Young’s modulus, *E*	73 GPa
Grain density, $\rho$	2200 kg/m$^3$
Poisson’s ratio, $\mu$	0.17
Tensile strength, $\sigma _{\text {N}}$	16 GPa
Shear strength, $\sigma _{\text {T}}$	9 GPa
Surface energy, $\gamma$	1.5 N/m
Restitution coefficient, $\eta$	0.9
Mean grain diameter, $d_{0}$	1.3 nm

Considering that a DEM particle is an order of magnitude larger than the bond lengths between atoms in silica (Si–O: 0.16 nm, O–O: 0.26 nm, Si–Si:0.31 nm) [33], Pham-Ba and Molinari [28] already show a significant reduction in computational time by using their DEM contact law compared to MD. To further reduce computational costs and enable modeling of larger domains, a FEM–DEM bridging coupling approach is deemed necessary.

### FEM–DEM coupling

The FEM–DEM bridging coupling involves an overlapping region $\Omega$ where the DEM and FEM are combined. This region, also referred to as the “bridging” zone, is where the two discretized schemes are reconciled. The methodology employed in this paper for FEM–DEM bridging coupling was previously described in Voisin-Leprince et al. [34]. Both strong and weak formulations were studied in the context of small deformations with Hertz contact between DEM particles. Considering the results of [34], a strong formulation and a force subtraction method at the FEM–DEM interface will be employed in this paper. Here, we provide a summary of the method (for more details, refer to [34]).

The first step in the coupling process involves defining an energy weighting that controls the influence of each domain. The global energy is then defined as a Hamiltonian:

1 $$\begin{aligned} H = \int _\Omega \alpha (X) E^{C}(X) + (1-\alpha (X))E^{D}(X) \quad dX. \end{aligned}$$

Here $E^{C}$ and $E^{D}$ are the energy densities in the continuum and discrete regions, respectively. These are weighted using a scaling function $\alpha (X)$ which defines the influence of the continuum domain for a given spatial location *X*. However, the energy weighting does not ensure kinematic consistency between the coupled models. To address this, Lagrange constraints are employed to link the two models. For a strong coupling, the constraint applied to the particles is expressed as:

2 $$\begin{aligned} {\varvec{\textrm{g}}} = \varvec{N}^T\varvec{u}-\varvec{d} = \varvec{0}. \end{aligned}$$

Here $\varvec{u}$ and $\varvec{d}$ denote the displacement of the nodes and particles, respectively, while $\varvec{N}$ represents the standard finite-element shape functions evaluated at the positions of each particle. This means that each particle is constrained to an interpolated position obtained from the finite element discretization. Both the energy weighing and the Lagrange constraint lead to a Lagrangian $H_{\textrm{L}}$:

3 $$\begin{aligned} H{\textrm{L}} = H+\varvec{\lambda }^{T}{} {\textbf {g}}, \end{aligned}$$

where *H* is the weighted Hamiltonian, and $\varvec{\lambda }$ the Lagrange multiplier vector. This modified Hamiltonian leads to new equations of motion:

4 $$\begin{aligned} \left\{ \begin{array}{ll} \overline{\varvec{M}}\varvec{\ddot{u}} = \overline{\varvec{{F}}} + \varvec{\lambda } \cdot \frac{\partial \varvec{\textrm{g}}}{\partial \varvec{u}} \\ \\ \overline{\varvec{m}}\varvec{\ddot{d}} = \overline{\varvec{{f}}} + \varvec{\lambda } \cdot \frac{\partial \varvec{\textrm{g}}}{\partial \varvec{d}} \end{array} . \right. \end{aligned}$$

In these equations, $\varvec{u}$, $\varvec{F}$, and $\varvec{M}$ represent the displacements, forces, and masses of the continuum domain, while $\varvec{d}$, $\varvec{f}$, and $\varvec{m}$ represent the displacements, forces, and masses of the discrete domain. Through energy weighing, the masses and forces are altered, giving rise to $\overline{\varvec{m}}$, $\overline{\varvec{f}}$ and $\overline{\varvec{M}}$, $\overline{\varvec{F}}$. Considering the Verlet time integration scheme [4] this results in solving:

5 $$\begin{aligned} \varvec{\dot{\textrm{g}}} = \varvec{\textrm{A}} \varvec{\lambda }, \end{aligned}$$

where $\varvec{\textrm{A}}$ is the constraint matrix, which is determined by the constraint function $\varvec{\textrm{g}}$. Therefore, the velocities of the nodes and particles in the overlapping region are corrected using the Lagrange multiplier vector $\varvec{\lambda }$.

The simulations are launched using the open-source *LibMultiScale* software [5–8] that realizes the coupling between the discrete and continuum regions. This software utilizes *Akantu* [3] for the continuum domain and *LAMMPS* [32] for the discrete domain. The contact law developed by Pham-Ba and Molinari [28] is incorporated in the *LAMMPS* version called by *LibMultiScale*.

## Results

**Fig. 1:**
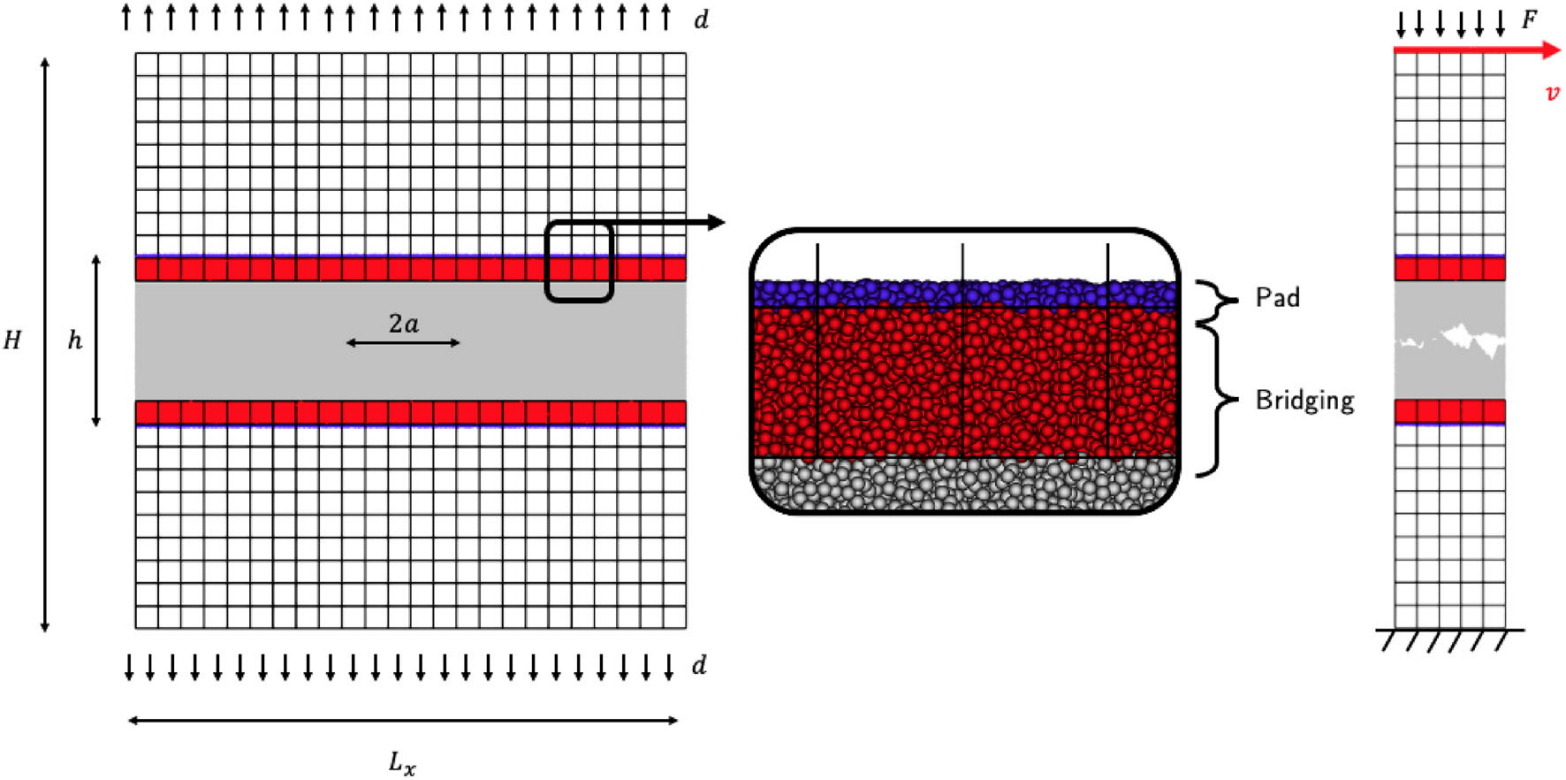
Left: scheme of the mode I crack propagation. Right: geometry of the shearing system. Middle: illustration of the overlapping zone of the coupling method, in red the bridging region where the coupling occurs, and in purple the pad region which serves as boundary for the DEM particles

The geometries of the simulated system are shown in Fig. 1. The left side illustrates the geometry of the mode I crack propagation simulation, while the right side shows the geometry of the shearing system that leads to wear debris formation. Both cases share common geometry parameters. The systems are quasi-2D, with a thickness in the *z* direction of $3d_{0}$. The FEM elements have a characteristic size in the *x* and *y* directions of $h_{\text {e}} = 20d_{0}$. The height of the bridging region (indicated in red in Fig. 1) is chosen to be equal to the height of one FEM element, $h_{\text {e}}=20d_{0}$. The pad region, indicated in purple in Fig. 1, serves as boundary conditions for the DEM particles by constraining all contained particles to the FEM displacement field. The height of the pad region is set to $h_{\text {pad}}=3d_{0}$. Periodic boundary conditions are applied in the *x* and *z* directions for both simulation setups.

**Fig. 2:**
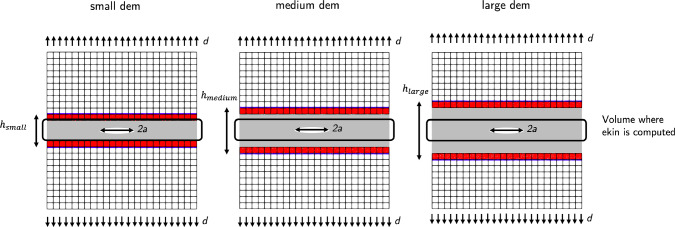
Schematic that illustrates the three DEM cases: small DEM ($110d_{0}$), medium DEM ($150d_{0}$), and large DEM ($190d_{0}$). The region where the kinetic energy is computed in each case is indicated by a black rectangle

### Mode I crack propagation

The mode I crack simulations were conducted on three coupled systems with a total height of $500d_{0}$, each having different DEM heights: small ($110d_{0}$), medium ($150d_{0}$), and large ($190d_{0}$). These DEM heights include the bridging and pad regions. Studying multiple DEM heights allows to assess whether the location of the coupling region/FEM region has any influence on the DEM behavior. To ensure a fair comparison of the coupling results, a pure DEM simulation of size $500d_{0}$ was also performed. For these simulations, a total length of $L_{x}=400d_{0}$ is considered. An imposed vertical displacement induces an opening of width 2*a* where the bonds between opposed particles in the upper and lower lips are disabled.

**Fig. 3:**
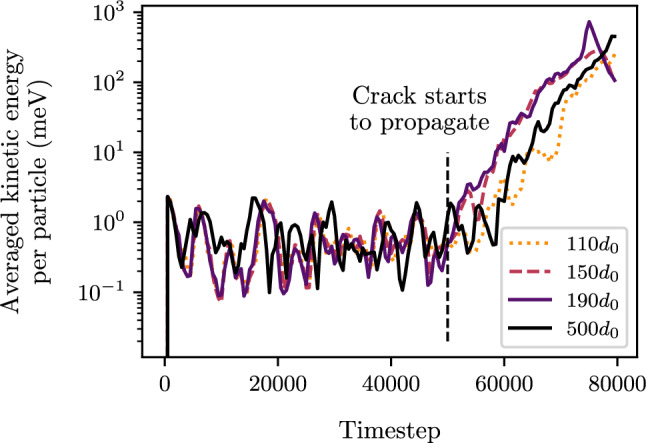
Kinetic energy per particle for the three coupled systems with initial DEM heights: $110d_{0}$, $150d_{0}$, $190d_{0}$ and for the pure DEM simulation $500d_{0}$. Total height of each simulation is $500d_{0}$

**Fig. 4:**
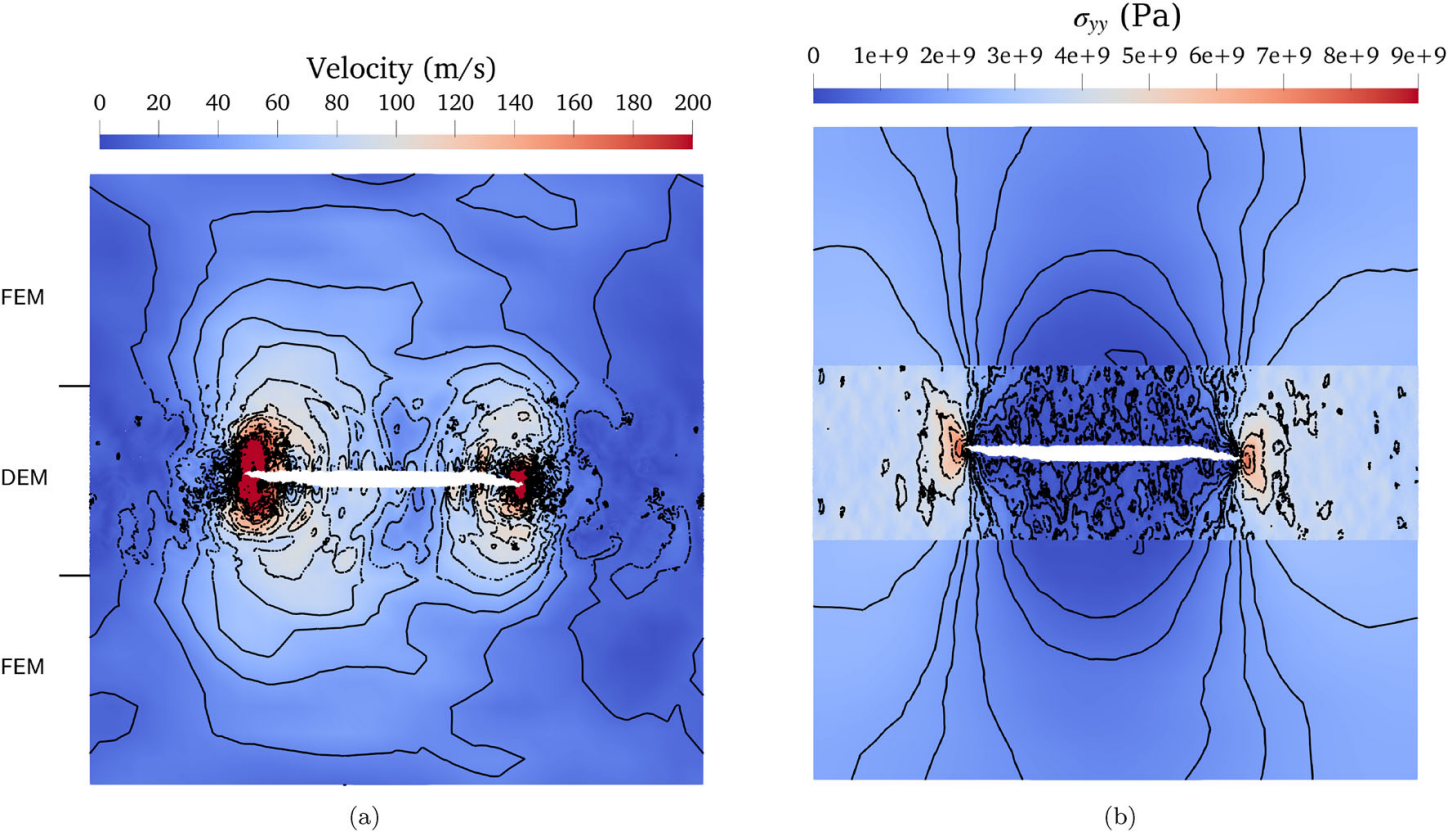
**a** Illustrates the velocity field, while **b** shows the stress field $\sigma _{yy}$ for the coupled system composed of the medium DEM ($150d_{0}$). The stress field is averaged within the DEM over a spherical volume with a radius of $r=6d_{0}$. Isolines for each field are represented in black. In (**a**), the top and bottom line elements have been omitted to enhance the visualization of crack propagation over the boundary limits. Furthermore, in (**a**), vertical black lines indicate the boundary between the top of the DEM and the FEM

To assess the consistency of the coupling results in mode I crack propagation, the kinetic energy per particle is evaluated. For each simulation, the kinetic energy is evaluated within the same region corresponding to the smallest DEM portion, excluding the bridging and pad regions (refer to Fig. 2).

The mode I crack propagation simulation is characterized by two stages. The first stage corresponds to the opening of the crack lips in the DEM region. Then, there is a second stage corresponding to the propagation of the crack. The kinetic energy during these two phases is represented in Fig. 3. It can be observed that the kinetic energy of the three coupled systems and the pure DEM simulation are in close agreement during both the opening phase and the crack propagation phase. Note that a perfect match cannot be obtained for an amorphous material due to a random organization of particles in parts of the simulation domain. This confirms that the crack initiation occurs at a similar time in each simulation. The similar results between the coupled systems and the pure DEM simulation validate the accuracy of the coupled systems in reproducing the crack propagation kinetics observed in the pure DEM simulation. Additionally, it shows that the location of the FEM region does not influence the DEM behavior.

To showcase the precision of the FEM–DEM coupling in mode I crack propagation, we visualized the velocity field. Figure 4a shows the velocity field for the medium DEM size case. In Fig. 4a, we observe a precise connection of the contour lines between the DEM and FEM, indicating a continuity of the velocity field from one domain to the other.

While studying crack propagation, stress concentration is a key aspect. In the FEM, the Cauchy stress tensor $\varvec{\sigma _{\text {FEM}}}$ is computed using the constitutive relation $\varvec{\sigma _{\text {FEM}}}= \varvec{C}:\varvec{\varepsilon }$, with $\varvec{\varepsilon }$ the infinitesimal strain tensors and $\varvec{C}$ the elastic modulus tensor. Whereas in the DEM, the stress $\varvec{\sigma _{\text {DEM}}}$ is computed using the virial stress (see LAMMPS documentation [15]). DEM stresses are averaged over a spherical volume, considering spherical radii of: $r=3d_{0}$, $r=6d_{0}$, $r=8d_{0}$, and $r=10d_{0}$. The largest radius considered leads to a diameter similar to the size of the FEM element. For a radius of $r=3d_{0}$, the stress is not averaged over a sufficiently large region, resulting in noisy results (see Figure 9a in the supplementary material). On the other hand, for a radius of $r=8d_{0}$ and $r=10d_{0}$, the stress is averaged over a region that is too extensive, leading to smoother results but blurring the stress concentration at the crack tips (see Figure 9b in the supplementary material which depicts the stress for a radius of $r=8d_{0}$; results for a radius of $r=10d_{0}$ are not shown because they are very similar to $r=8d_{0}$). Therefore, for representing the stress, we have chosen a radius of $r=6d_{0}$, which provides a notable continuity of the isolines between the FEM and DEM domains (see Fig. 4b). Thus, averaging the DEM stress over a properly chosen region leads to comparable results in FEM and DEM. Consequently, our approach can be used for a comprehensive study of crack propagation.

Table 2 presents the time duration for each simulation timestep $\Delta t$ in the three coupled simulations (DEM height: $110d_{0}$, $150d_{0}$, $190d_{0}$) and the pure DEM simulation (DEM height: $500d_{0}$). The computation of the time per timestep was performed using the serial version of *LibMultiScale* and *LAMMPS*. As expected, it can be observed that the time per timestep is smaller in the coupled simulations compared to the pure DEM simulation. Additionally, the time per timestep increases with the number of particles *N*. Therefore, the FEM–DEM coupling accurately represents the behavior expected from a pure DEM simulation, while significantly reducing computational time.

**Table 2:** Time per simulation timestep for the mode I crack propagation simulations.

DEM height	*N*	Time per $\Delta t$ (s)
$110d_{0}$	229120	1.71
$150d_{0}$	312360	2.14
$190d_{0}$	395800	2.57
$500d_{0}$	1243050	10.23

*N* is the number of particles and, $\Delta t$ the timestep

### Surface wear during relative sliding

For the shearing systems, a total length of $L_{x}=100d_{0}$ is considered, and rough surfaces are introduced in the DEM. The roughness is characterized by two main parameters: the Hurst exponent $\mathcal {H}$ and the arithmetical mean deviation of heights $S_{\textrm{a}}$, with chosen values for this study being $\mathcal {H}=0.8$ [24] and $S{\textrm{a}} = 5d_{0}$. The choice of $S{\textrm{a}}$ is based on the critical junction size $d^*$ which represents the size at which two surfaces in shear motion begin to detach and form wear particles [2]. Previous research by Pham-Ba and Molinari [28] has demonstrated that, based on their contact law and the material properties of SiO$_2$, $d^*$ falls within the range of 10–20 nm. Thus, to facilitate wear formation using $d^*$ as a reference, we select an arithmetical mean deviation of $S{\textrm{a}} = 5d_{0}$, resulting in a DEM junction at the start of the simulation of size $d^*$. The DEM is carved to the desired roughness using Tamaas code [19]. In the simulation, the system is blocked at the bottom boundary. The top nodes of the FEM are subjected to a constant normal pressure of 100 MPa and a constant shearing velocity of $v_\textrm{s} = 0.01 \sqrt{E/\rho }$.

To study the formation of wear particles, we considered three coupled systems, each with a total height of $500d_{0}$, and different DEM heights: $110d_{0}$, $150d_{0}$, $190d_{0}$. These DEM heights account for both the bridging and pad regions. To make a proper comparison of the results obtained from the coupling, a pure DEM simulation of size $500d_{0}$ was also performed. Similarly to the mode I crack propagation, the kinetic energy for each case was computed within the same specific region characterized by the smallest DEM height, excluding the bridging and pad regions.

**Fig. 5:**
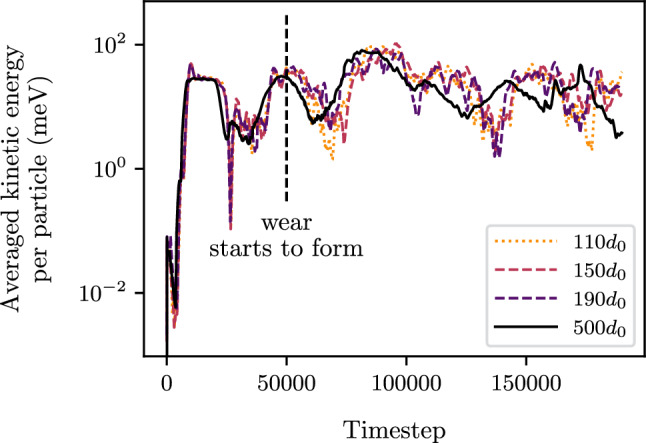
Kinetic energy per particle for the five coupled systems with initial DEM heights: $110d_{0}$, $150d_{0}$, $190d_{0}$ and for the pure DEM simulation $500d_{0}$. Total height of each simulation is $500d_{0}$

The coupling simulations can be divided into three stages: uniform deformation before crack formation, debris formation, and subsequent rolling wear. Figure 5 shows the kinetic energy evolution of the coupled and pure DEM systems. It can be observed that the kinetic energy maintains a consistent level throughout the simulation. Additionally, both the coupled system’s kinetic energy and that of the pure DEM simulation closely align. This confirms that the coupled system accurately captures the velocity field, regardless of the location of the coupling/FEM region.

**Fig. 6:**
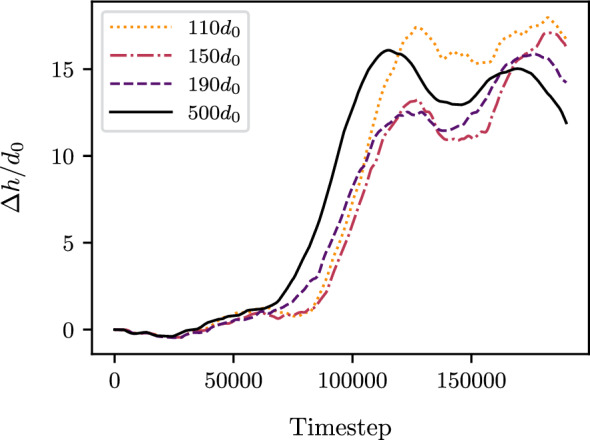
Evolution of the domain thickness $\Delta h$ over time, normalized by the mean grain diameter $d_{0}$. The evolution of the domain thickness was computed for the five coupled systems with initial DEM heights: $110d_{0}$, $150d_{0}$, $190d_{0}$ and for the pure DEM simulation $500d_{0}$. Total height of each simulation is $500d_{0}$

To confirm that the coupling is accurately representing the expected physics of the pure DEM, we examined the evolution of the whole domain thickness over time. Figure 6 shows this evolution, with $\Delta h = h_{t} - h_{0}$ the change in the domain thickness through time, $h_{t}$ being the total domain thickness at time *t*, and $h_{0}$ the total domain thickness at the initial time step. The evolution of the domain thickness, over time, can be interpreted as the size of the created wear debris. Figure 6 demonstrates that both the coupled systems and the pure DEM simulation initiate debris formation at a similar time (at around 50, 000 timesteps), with the debris reaching a comparable size of approximately $15d_{0}$. Thus, the coupling approach effectively captures the wear debris physics of the pure DEM simulation. Furthermore, the location of the FEM region does not affect wear formation. In other words, launching the simulation with the smallest DEM coupled system is sufficient to represent the wear debris physics of the pure DEM simulation while reducing computational cost.

**Fig. 7:**
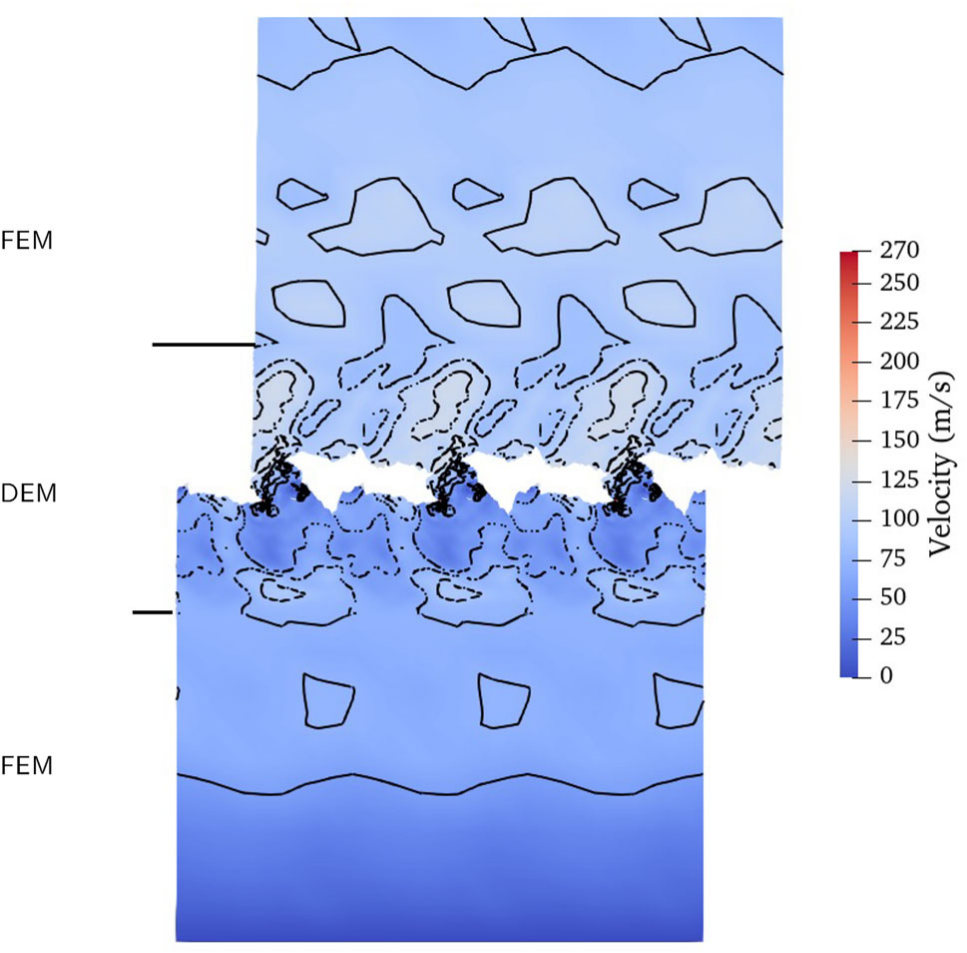
Representation of the velocity field for the coupled system with a DEM height of 150$d_{0}$. The isolines of the velocity field are indicated in black. Vertical black lines denote the boundary between the top of the DEM and the FEM

To illustrate the precision of the coupling in the case of third body creation, we visualized the velocity field for an initial DEM height of $150d_0$ (Fig. 7). The wear formation process introduces perturbations in the velocity field, which can be observed in Fig. 7. Importantly, we observe a precise connection of the velocity field and contour lines between the DEM and FEM, indicating that the velocity is accurately transmitted from one domain to the other. This confirms the high level of precision achieved by the coupling. Furthermore, we analyzed the stress field to visualize the crack propagation during the wear formation process (see Figure ref 8). Similar to the mode I crack simulation, we calculated the average stress of particles within a sphere of radius $6d_0$.

**Fig. 8:**
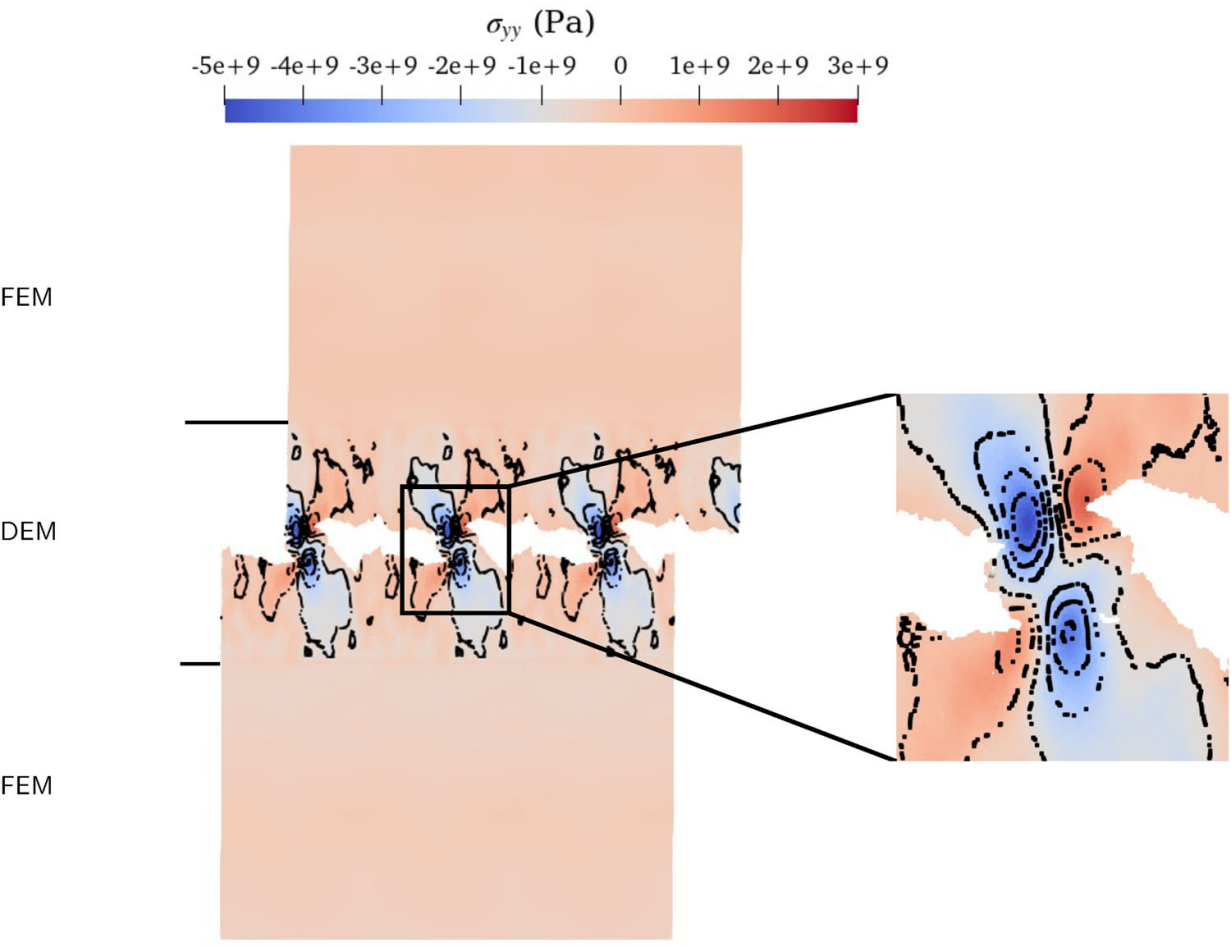
Representation of the stress field $\sigma _{yy}$ for the coupled system with a DEM height of 150$d_{0}$. The isolines of the stress field are indicated in black. Vertical black lines denote the boundary between the top of the DEM and the FEM. On the right, a zoomed-in view of the wear debris is presented

Table 3 presents the time duration for each simulation timestep $\Delta t$ in the five coupled simulations (DEM height: $110d_{0}$, $150d_{0}$, $190d_{0}$) and the pure DEM simulation (DEM height: $500d_{0}$). Similar to the mode I simulations, the time per timestep was computed using the serial version of *LibMultiScale* and *LAMMPS*. Consistent with the mode I results, the time per timestep is smaller in the coupled simulations compared to the pure DEM simulation, and it increases with the number of particles *N*.

**Table 3:** Time per simulation timestep for the wear formation simulations.

DEM height	N	Time per $\Delta t$ (s)
$110d_{0}$	42039	0.17
$150d_{0}$	58687	0.22
$190d_{0}$	75375	0.28
$500d_{0}$	206481	0.55

*N* is the number of particles and, $\Delta t$ is the timestep

## Conclusion

In this exploratory study, we have demonstrated the effectiveness of the FEM–DEM bridging coupling [34] in simulating discontinuous events involving material failure. We investigated two scenarios: mode I crack propagation and wear debris creation during the contact between sliding rough surfaces.

For mode I crack propagation, we observed that crack initiation occurred simultaneously in both the coupled system and the pure DEM system. Furthermore, by exploring multiple DEM heights in the coupled simulations, we confirmed that the location of the coupling/FEM region does not influence crack propagation.

In the case of wear simulations, we observed that wear initiation and the resulting debris size were similar for both the coupled systems and the pure DEM system. Similarly to mode I crack propagation, studying various DEM heights in the coupled simulations shows that the location of the coupling/FEM region has no impact on wear formation.

In summary, we have effectively demonstrated that the coupling approach accurately captures the crack propagation and wear formation physics of the pure DEM. It is worth noting that the coupling region was intentionally positioned at a sufficient distance from the DEM region undergoing large deformation to prevent undesired crack propagation into the bridging region. Given that it is challenging for the FEM to precisely model the inelastic deformation of the DEM, such an arrangement would lead to a physically inaccurate representation.

## Supplementary Information

Below is the link to the electronic supplementary material.

Supplementary file 1 (pdf 1847 KB)

## Data Availability

Dataset corresponding to the paper: http://dx.doi.org/10.5281/zenodo.11654613.
