# Supplementary material for: Two-scale concurrent simulations for crack propagation using FEM–DEM bridging coupling
Source: Comput Part Mech. 2024 Jul 27;11(5):2235–43. doi: 10.1007/s40571-024-00788-x (PMC11442508; doi:10.1007/s40571-024-00788-x)
Supplement: Supplementary file 1 — (pdf 1847 KB) [file 40571_2024_788_MOESM1_ESM.pdf]

Supplementary material

Contact law from Pham-Ba and Molinari [28]

The force $\boldsymbol{F}$ acting between a given pair of particles is the sum of a normal component F_N, a tangential component F_T, and velocity damping components $c_\mathrm{N}v_\mathrm{N}$ and $c_\mathrm{T}v_\mathrm{T}$, where v_N and v_T are the relative velocities in the normal and tangential directions, and c_N and c_T are the damping factors. We note $\boldsymbol{n}_\mathrm{N}$ and $\boldsymbol{n}_\mathrm{T}$ the units vectors in the normal and tangential directions:

$$\boldsymbol{F} = -(F_\mathrm{N} + c_\mathrm{N}v_\mathrm{N})\boldsymbol{n}_\mathrm{N} - (F_\mathrm{T} + c_\mathrm{T}v_\mathrm{T})\boldsymbol{n}_\mathrm{T} \tag{6}$$

The normal component depends on the normal overlap δ_N between the particles and is expressed as:

$$F_\mathrm{N} = \begin{cases} k_\mathrm{N}\delta_\mathrm{N} & \text{if } \delta_\mathrm{N} \leq \delta_\mathrm{e}, \\ -\dfrac{k_\mathrm{N}\delta_\mathrm{e}}{\delta_\mathrm{f} - \delta_\mathrm{e}}(\delta_\mathrm{N} - \delta_\mathrm{f}) & \text{if } \delta_\mathrm{e} < \delta_\mathrm{N} \leq \delta_\mathrm{f}, \\ 0 & \text{if } \delta_\mathrm{N} > \delta_\mathrm{f}, \end{cases} \tag{7}$$

with k_N the Hookean stiffness, δ_e the maximum elastic distance, and δ_f the fracture distance. The tangential force depends on the sliding distance δ_T and is defined as:

$$F_\mathrm{T} = \min(k_\mathrm{T}\delta_\mathrm{T}, F'_\mathrm{m,T}), \tag{8}$$

$$F'_\mathrm{m,T} = \min\left(\frac{\delta_\mathrm{f} - \delta_\mathrm{N}}{\delta_\mathrm{f}}, 1\right) F_\mathrm{m,T}, \tag{9}$$

with $F_\mathrm{m,T}$ the maximum tangential force, and k_T the tangential stiffness. The inter-particle force parameters between particles are determined based on desired material properties using the following relations:

$$m_\mathrm{eff} = \frac{m_i m_j}{m_i + m_j}, \qquad r_\mathrm{eff} = \min(r_i, r_j) \tag{10}$$

$$A_\mathrm{N} = \sqrt{2}r_\mathrm{eff}^2\frac{1}{1-2\nu}, \qquad A_\mathrm{T} = \sqrt{2}r_\mathrm{eff}^2\frac{1-4\nu}{(1-2\nu)(1+\nu)}, \tag{11}$$

$$k_\mathrm{N} = \frac{A_\mathrm{N}E}{r_i + r_j}, \qquad k_\mathrm{T} = \frac{A_\mathrm{T}E}{r_i + r_j}, \tag{12}$$

$$c_\mathrm{N} = \frac{2(1-\eta)}{\pi}\sqrt{k_\mathrm{N}m_\mathrm{eff}}, \qquad c_\mathrm{T} = \frac{2(1-\eta)}{\pi}\sqrt{k_\mathrm{T}m_\mathrm{eff}}, \tag{13}$$

$$\delta_\mathrm{e} = \frac{(r_i + r_j)\sigma_\mathrm{N}}{E}, \qquad \delta_\mathrm{f} = \frac{4\gamma}{\sigma_\mathrm{N}}, \tag{14}$$

$$F_\mathrm{m,\,T} = A_\mathrm{T}\sigma_T, \tag{15}$$

with r_i and r_j the radii of the interacting particles, m_i and m_j their masses, E the Young's modulus, σ_N the tensile strength, σ_T the shear strength, γ the surface energy, and η the restitution coefficient. These relations are valid for radii of interacting particles, which respect:

$$r_i + r_j \leq d_\mathrm{c}, \text{ with the critical diameter } d_\mathrm{c} = \frac{4\gamma E}{\sigma_\mathrm{N}^2}. \tag{16}$$

Additionally, if a particle is smaller than the neighbor size δ_f, it will interact with particles beyond its closest neighbors, extending the interaction range. Therefore, to avoid an important increase in computational cost, the minimum particle's size should be:

$$d_{\min} = \frac{4\gamma}{\sigma_\mathrm{N}} \tag{17}$$

Mode I crack propagation

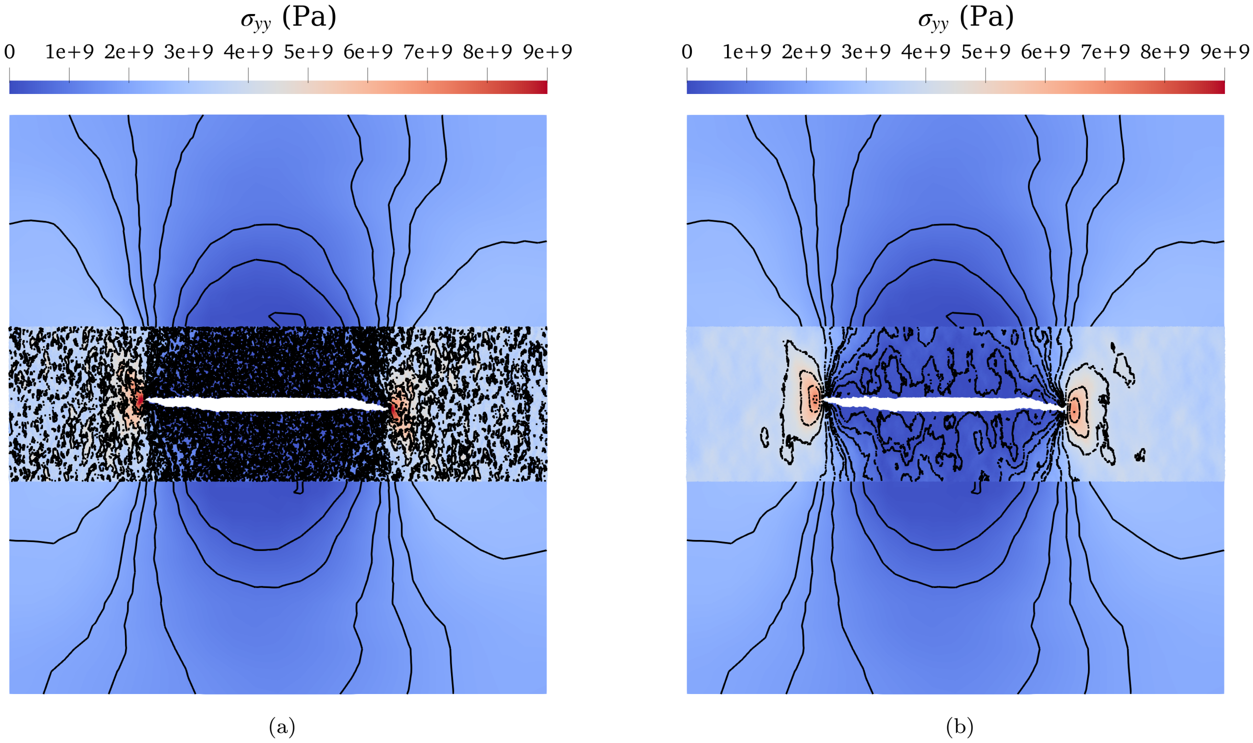

Figure 9: (a.) and (b.) represents the stress field σ_{yy} for the coupled system composed of the medium DEM ($150d_0$). In (a), the stress field is averaged over a spherical volume of radius $r = 3d_0$ within the DEM, while in (b), the stress field is averaged over a spherical volume of radius $r = 8d_0$. The isolines of the stress field are depicted in black.
